# Diagnostic Accuracy of Liver and Spleen Elastography for Detecting Portal Hypertension Defined by Hepatic Venous Pressure Gradient (HVPG) in Chronic Liver Disease: A Systematic Review

**DOI:** 10.7759/cureus.110383

**Published:** 2026-06-07

**Authors:** Mohammed Saleem Yousuf, Khine Tun, Philip D Baalaboore, Mehak G Mastoi, Ursala Adil, Ahmad Elsaid, Mehnaz R Muna, Vignesh Ramachandran, Nabeeha Azhar, Rabia Azhar, Ahmed Raza Bhutta

**Affiliations:** 1 Internal Medicine, Viswabharathi Medical College, Kurnool, IND; 2 Gastroenterology and Hepatology, Oxford University Hospitals NHS Foundation Trust, Oxford, GBR; 3 Internal Medicine, Royal Hampshire County Hospital, Winchester, GBR; 4 Paediatrics, Ledzokuku-Krowor Municipal Assembly (LEKMA) Hospital, Accra, GHA; 5 Geriatrics, Montefiore Medical Center (Wakefield Campus), Bronx, USA; 6 Internal Medicine, Interfaith Medical Center, New York, USA; 7 Internal Medicine, Trinitas Regional Medical Center, Elizabeth, USA; 8 Internal Medicine, RWJBarnabas Health, West Orange, USA; 9 Gastroenterology, University Hospital Birmingham NHS Foundation Trust, Birmingham, GBR; 10 Internal Medicine, Melmaruvathur Adhiparasakthi Institute of Medical Sciences and Research, Melmaruvathur, IND; 11 Internal Medicine, Avicenna Medical College, Lahore, PAK; 12 Internal Medicine, Lahore Medical and Dental College, Lahore, PAK; 13 Internal Medicine, Rawalpindi Medical University, Rawalpindi, PAK

**Keywords:** chronic liver disease, diagnostic accuracy, hepatic venous pressure gradient, liver stiffness measurement, portal hypertension, shear-wave elastography, spleen stiffness

## Abstract

Portal hypertension is a major consequence of chronic liver disease and a key determinant of clinical outcomes, traditionally assessed using the invasive hepatic venous pressure gradient (HVPG). Given the limitations of HVPG measurement, ultrasound-based elastography has emerged as a promising noninvasive alternative. This systematic review evaluated the diagnostic accuracy of liver stiffness measurement (LSM) and spleen stiffness measurement (SSM), obtained through transient elastography (TE) and shear-wave elastography (SWE), for detecting HVPG-defined portal hypertension in adults with chronic liver disease. A comprehensive search of PubMed/MEDLINE, Embase, and the Cochrane Library, from database inception through December 2024, identified eight eligible studies comprising 1,636 patients. Clinically significant portal hypertension was primarily defined as an HVPG of ≥10 mmHg. Across studies, LSM demonstrated moderate-to-high discriminatory performance, with area under the receiver operating characteristic curve (AUROC) values ranging from 0.74 to 0.94, depending on modality and population. SWE generally showed superior performance compared with conventional TE, particularly when standardized reliability criteria were applied. SSM provided complementary hemodynamic information and improved risk stratification in selected cohorts, although it did not consistently outperform LSM alone. Variability in stiffness cutoffs and study design contributed to methodological heterogeneity. Overall, ultrasound-based elastography demonstrates clinically meaningful diagnostic accuracy for identifying portal hypertension and offers a practical, noninvasive approach for risk stratification, particularly in compensated cirrhosis; however, it does not fully replace invasive HVPG measurement.

## Introduction and background

Portal hypertension is a central pathophysiological consequence of chronic liver disease (CLD) and cirrhosis, arising from increased intrahepatic vascular resistance and progressive architectural distortion of the hepatic parenchyma. As portal pressure increases, patients become predisposed to clinically significant complications, including ascites, variceal hemorrhage, hepatic encephalopathy, and progressive decompensation [1]. The degree of portal hypertension is therefore not only a marker of disease severity but also a determinant of prognosis and therapeutic strategy. Clinically significant portal hypertension (CSPH), commonly defined as a hepatic venous pressure gradient (HVPG) of ≥10 mmHg, identifies patients at increased risk of developing varices and other complications, while higher thresholds are associated with an increased risk of bleeding and poorer clinical outcomes [2,3].

Measurement of HVPG remains the reference standard for assessing portal pressure. It provides direct hemodynamic quantification and has been extensively validated for prognostic stratification and therapeutic monitoring. However, HVPG measurement is invasive, resource-intensive, and available only in specialized centers. These limitations restrict its routine use in clinical practice, particularly for screening and longitudinal monitoring. Consequently, there has been sustained interest in developing reliable, noninvasive methods capable of estimating portal pressure and identifying clinically relevant thresholds [4].

Ultrasound-based elastography techniques, including transient elastography (TE), point shear-wave elastography (pSWE), and two-dimensional shear-wave elastography (2D-SWE), have emerged as promising tools for noninvasive assessment. Liver stiffness measurement (LSM) has been widely studied as a surrogate marker of hepatic fibrosis and has demonstrated moderate correlation with portal pressure [5,6]. More recently, spleen stiffness measurement (SSM) has gained attention, reflecting the hemodynamic and structural changes occurring in the splenic circulation in the setting of portal hypertension. Several prospective and retrospective diagnostic studies have evaluated the relationship between LSM or SSM and HVPG-defined portal hypertension, reporting variable degrees of correlation and diagnostic performance. However, heterogeneity in study design, patient populations, elastography modalities, and diagnostic thresholds has resulted in inconsistent estimates of accuracy [7,8].

Although individual studies suggest that ultrasound-based elastography may have clinical utility in detecting portal hypertension, uncertainty remains regarding its overall diagnostic performance when assessed against the invasive gold standard. Furthermore, the relative performance of liver versus spleen stiffness, as well as the consistency of findings across different elastography techniques, warrants systematic evaluation.

The objective of this systematic review is to evaluate the diagnostic accuracy of ultrasound-based elastography, specifically liver stiffness measurement and spleen stiffness measurement, for detecting portal hypertension in adults with chronic liver disease, using hepatic venous pressure gradient (HVPG) as the reference standard. By synthesizing the primary evidence available, this review aims to provide a comprehensive assessment of the performance of these noninvasive modalities and to clarify their potential role in the clinical stratification of portal hypertension.

## Review

Materials and methods

Study Design and Reporting Framework

This study was conducted as a systematic review of diagnostic test accuracy to evaluate the performance of ultrasound-based elastography for detecting portal hypertension in adults with chronic liver disease, using hepatic venous pressure gradient (HVPG) as the reference standard. The methodology was developed in accordance with the Preferred Reporting Items for Systematic Reviews and Meta-Analyses (PRISMA) 2020 guidelines [9], and the review process adhered to established methodological standards for diagnostic accuracy studies. The objective was to synthesize available primary evidence assessing the ability of liver stiffness measurement (LSM) and spleen stiffness measurement (SSM) to identify HVPG-defined portal hypertension, particularly clinically significant portal hypertension (CSPH).

PICO Framework

The research question was structured according to the Population, Index test, Comparator, and Outcome (PICO) framework [10], adapted for diagnostic test accuracy studies. The population comprised adults with chronic liver disease or cirrhosis who underwent invasive HVPG measurement. The index test included ultrasound-based elastography techniques, namely transient elastography (TE), point shear-wave elastography (pSWE), and two-dimensional shear-wave elastography (2D-SWE), used to assess liver stiffness and/or spleen stiffness. The comparator (reference standard) was an HVPG measurement obtained via invasive hemodynamic assessment.

Eligibility Criteria

Studies were eligible for inclusion if they evaluated adult patients with chronic liver disease or cirrhosis who underwent both ultrasound-based elastography and HVPG measurement and if they reported diagnostic performance metrics or sufficient data to determine diagnostic accuracy. Prospective and retrospective cohort studies, multicenter validation studies, and diagnostic accuracy studies were considered. Studies were excluded if they did not use HVPG as the reference standard, did not report diagnostic performance outcomes, focused exclusively on pediatric populations, or evaluated imaging modalities other than ultrasound-based elastography. Only original research articles were included in the synthesis.

Information Sources and Study Selection

Relevant studies were identified through structured electronic searches of PubMed/MEDLINE, Embase, and the Cochrane Library from database inception through December 2024. The search strategy combined Medical Subject Headings (MeSH) and free-text terms using Boolean operators to maximize sensitivity and specificity. Core search terms included “portal hypertension," “hepatic venous pressure gradient," “HVPG,” "elastography," “transient elastography," “shear wave elastography," “liver stiffness," and “spleen stiffness." These were combined using operators such as (“portal hypertension” OR “hepatic venous pressure gradient” OR HVPG) AND (“elastography” OR “transient elastography” OR “shear wave elastography” OR “liver stiffness” OR “spleen stiffness”). Controlled vocabulary terms such as the MeSH headings “Hypertension, Portal” and “Elasticity Imaging Techniques” were incorporated where applicable. Searches were limited to human studies involving adult populations, and no initial language restrictions were applied during screening. Titles and abstracts were independently screened for relevance, followed by full-text review based on predefined eligibility criteria. Study selection was conducted in accordance with PRISMA recommendations, and discrepancies in eligibility assessment were resolved through discussion and consensus to ensure methodological rigor and minimize selection bias.

Data Extraction

Data were extracted using a standardized approach to ensure consistency and completeness. Extracted variables included study characteristics such as author, year, country, design, sample size, patient population, and underlying etiology of liver disease. Technical parameters included elastography modality, stiffness parameter assessed (liver and/or spleen), HVPG threshold definitions, stiffness cut-off values, and reported diagnostic performance metrics, including AUROC, sensitivity, specificity, predictive values, accuracy, and correlation coefficients. When multiple thresholds were reported, those corresponding to CSPH were preferentially recorded.

Quality Assessment and Risk of Bias

Methodological quality and risk of bias were evaluated using the Quality Assessment of Diagnostic Accuracy Studies (QUADAS-2) tool [11]. This framework assessed four domains: patient selection, index test, reference standard, and flow and timing. Each domain was evaluated for risk of bias, and the first three domains were additionally assessed for concerns regarding applicability. Studies were categorized as low, moderate, or high risk of bias based on reporting transparency, blinding, selection methodology, and completeness of outcome data. The quality assessment informed the interpretation of heterogeneity and overall confidence in the synthesized findings.

Data Synthesis and Analysis

Given variability in study design, elastography modality, stiffness thresholds, and reported performance metrics, a structured qualitative synthesis was performed. Diagnostic performance was summarized descriptively, with emphasis on AUROC values and reported accuracy metrics for detecting CSPH. Where available, comparative patterns between elastography modalities and between liver and spleen stiffness were analyzed narratively. Heterogeneity was interpreted in the context of clinical spectrum differences, technical variability, and risk-of-bias domains. The synthesis focused on identifying consistent patterns in diagnostic discrimination and contextual factors influencing performance.

Ethical Considerations

As this study was a systematic review of previously published data, no direct patient involvement occurred, and ethical approval was not required. The review synthesized existing evidence to inform clinical decision-making and future research directions in non-invasive assessment of portal hypertension.

Results

Study Selection Process

**Figure 1:**
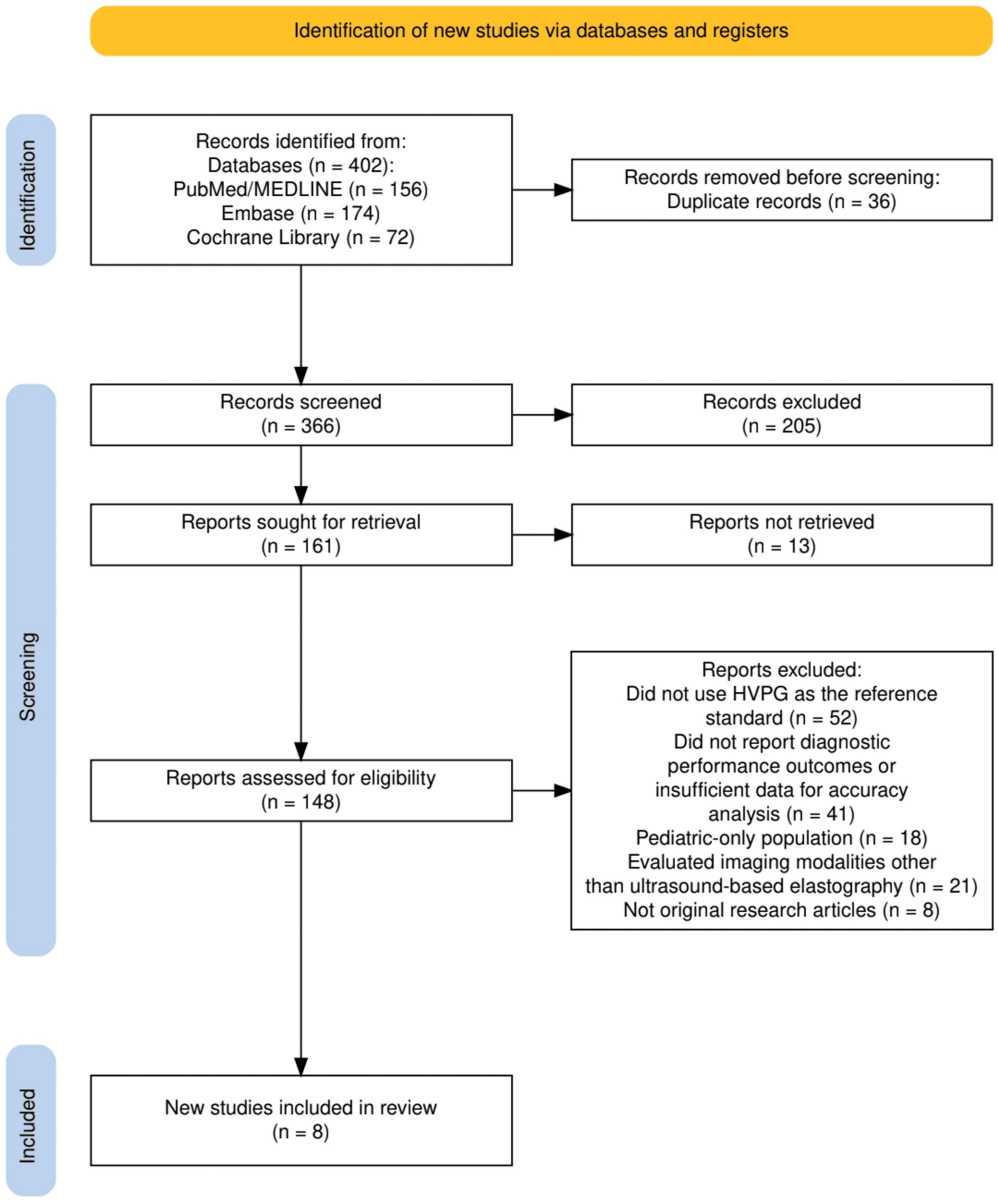
PRISMA 2020 flow diagram illustrating the study selection process. PRISMA: Preferred reporting items for systematic reviews and meta-analyses

The study selection process is illustrated in Figure 1. A total of 402 records were identified through electronic searches of PubMed/MEDLINE, Embase, and the Cochrane Library. After the removal of 36 duplicate records, 366 titles and abstracts were screened for relevance. Of these, 205 records were excluded based on predefined criteria. A total of 161 reports were sought for full-text retrieval, of which 13 could not be obtained, leaving 148 articles assessed for eligibility. Following full-text review, 140 studies were excluded due to not using HVPG as the reference standard, absence of diagnostic performance data, exclusive pediatric populations, evaluation of non-ultrasound imaging modalities, or non-original study design. Ultimately, eight studies met all inclusion criteria and were included in the final qualitative synthesis.

Characteristics of the Selected Studies

The characteristics of the included studies are summarized in Table 1. The eight selected studies comprised a total of 1,636 adult patients with chronic liver disease or cirrhosis undergoing concurrent ultrasound-based elastography and HVPG measurement. Study designs included predominantly prospective cohorts, along with retrospective and multicenter diagnostic validation studies, reflecting a mixture of single-center and international settings. The study populations represented diverse etiologies, including viral hepatitis, alcoholic liver disease, and nonalcoholic steatohepatitis, with most cohorts focusing on compensated or advanced cirrhosis. Elastography modalities varied across studies and included transient elastography, point shear-wave elastography, and two-dimensional shear-wave elastography, with assessments of liver stiffness alone or combined liver and spleen stiffness. HVPG thresholds were primarily centered on clinically significant portal hypertension, defined as ≥10 mmHg, although some studies additionally evaluated higher thresholds for severe portal hypertension. Reported diagnostic performance metrics included AUROC values, overall accuracy, predictive values, and correlation coefficients, demonstrating variability in cut-off thresholds and performance across modalities and patient populations.

**Table 1:** Characteristics of included studies evaluating liver and spleen elastography for detecting clinically significant portal hypertension. HVPG: Hepatic venous pressure gradient, LSM: Liver stiffness measurement, SSM: Spleen stiffness measurement, CSPH: Clinically significant portal hypertension, TE: Transient elastography, 2D-SWE: Two-dimensional shear wave elastography, MRE: Magnetic resonance elastography, AUROC: Area under the receiver operating characteristic curve, PPV: Positive predictive value, NPV: Negative predictive value, CI: Confidence interval

Study (Year)	Country	Design	N	Population	Elastography Modality	Stiffness Assessed	HVPG Definition	Cut-off (kPa)	AUROC / Accuracy
Kumar et al., 2017 [12]	India	Retrospective	326	Cirrhosis	TE	LSM	≥10 mmHg	21.6	0.74
Elkrief et al., 2015 [13]	France	Prospective	79	Advanced cirrhosis	SWE	LSM / SSM	≥10 mmHg	24.6 (LSM)	0.87 (LSM)
Rabiee et al., 2022 [14]	Multicenter	Diagnostic validation	222	NASH cirrhosis	TE	LSM-based model	≥10 mmHg	Model-based	>0.80
Zykus et al., 2015 [15]	Lithuania	Prospective	107	Chronic liver disease	TE	LSM / SSM	CSPH & SPH	17.4 (LSM)	88.7% accuracy
Jansen et al., 2017 [16]	Multicenter (Europe)	Prospective	158	Cirrhosis	Shear-Wave Elastography (SWE)	Liver & Spleen	≥10 mmHg (CSPH)	24.6 (L-SWE); 26.3 (S-SWE)	NR
Procopet et al., 2015 [17]	Spain	Prospective	88	Cirrhosis undergoing HVPG	Real-Time Shear-Wave Elastography (RT-SWE)	Liver (± Spleen)	≥10 mmHg (CSPH)	15.4 (LS RT-SWE)	0.939 (reliable measurements)
Colecchia et al., 2012 [18]	Italy	Prospective	100	HCV-related cirrhosis	Transient Elastography (TE)	Spleen & Liver Stiffness	HVPG measured (PH grading)	NR	R² = 0.85 (SS+LS model)
Ryu et al., 2020 [19]	South Korea	Retrospective	556	Alcoholic & Viral cirrhosis	Transient Elastography (TE)	Liver Stiffness (LSM)	≥10 mmHg; ≥12 mmHg	32.2 (≥10); 36.6 (≥12)	PPV 94.5% (≥10); 91.0% (≥12)

Quality Assessment

The methodological quality assessment of the included studies is summarized in Table 2. Overall, most studies demonstrated low risk of bias in the reference standard domain, as HVPG measurement was consistently applied and appropriately interpreted. The index test domain was generally rated as low to moderate risk, with some studies lacking detailed reporting of blinding procedures or predefined stiffness cut-offs. Patient selection presented moderate concerns in several studies, primarily due to retrospective designs, non-consecutive enrollment, or limited reporting on sampling strategies, which may introduce selection bias. The flow and timing domain showed moderate variability, particularly where the interval between elastography and HVPG measurement was not clearly specified or where incomplete data reporting was noted. Applicability concerns were largely low across studies, as the populations, index tests, and reference standards were aligned with the review question. Collectively, the quality assessment indicates that while the overall evidence base is methodologically sound, certain design and reporting limitations may contribute to heterogeneity in diagnostic performance estimates.

**Table 2:** Diagnostic performance and risk of bias assessment of elastography modalities for predicting portal hypertension. QUADAS-2: Quality Assessment of Diagnostic Accuracy Studies 2, HVPG: Hepatic venous pressure gradient, LSM: Liver stiffness measurement, SSM: Spleen stiffness measurement, CSPH: Clinically significant portal hypertension, AUROC: Area under the receiver operating characteristic curve, TE: Transient elastography, SWE: Shear wave elastography

Study (Year)	Patient Selection	Index Test	Reference Standard	Flow & Timing	Overall Risk of Bias	Applicability Concern
Kumar et al., 2017 [12]	Moderate	Low	Low	Moderate	Moderate	Low
Elkrief et al., 2015 [13]	Low	Low	Low	Low	Low	Low
Rabiee et al., 2022 [14]	Moderate	Moderate	Low	Low	Moderate	Moderate
Zykus et al., 2015 [15]	Low	Low	Low	Low	Low	Low
Jansen et al., 2017 [16]	Low	Low	Low	Moderate	Low–Moderate	Low
Procopet et al., 2015 [17]	Low	Moderate	Low	Moderate	Moderate	Low
Colecchia et al., 2012 [18]	Low	Low	Low	Low	Low	Low
Ryu et al., 2020 [19]	Moderate	Moderate	Low	Moderate	Moderate	Low

Discussion

Across the included studies, ultrasound-based elastography demonstrated consistent, though variable, ability to identify HVPG-defined portal hypertension in patients with chronic liver disease. Liver stiffness measurement generally showed moderate-to-high discriminatory performance for detecting clinically significant portal hypertension (CSPH), with area under the curve values ranging from approximately 0.74 to above 0.90 depending on modality and study population. In particular, prospective shear-wave elastography studies such as those by Elkrief et al. [13] and Procopet et al. [17] reported strong diagnostic performance under standardized measurement conditions, while large retrospective cohorts like Kumar et al. [12] and Ryu et al. [19] supported the clinical utility of transient elastography with etiology-specific cut-offs. Multicenter data from Jansen et al. [16] further suggested that combining liver and spleen stiffness may enhance rule-in and rule-out strategies for CSPH. Collectively, these findings indicate that ultrasound-based elastography provides a clinically meaningful, non-invasive approximation of portal pressure, although performance varies according to modality, population characteristics, and measurement reliability criteria.

A consistent pattern across the included studies is that liver stiffness measurement (LSM) demonstrates moderate to high diagnostic performance for detecting clinically significant portal hypertension (CSPH), though performance varies by modality and clinical context. Shear-wave elastography (SWE), particularly when reliability criteria are applied, tended to yield higher discriminatory ability compared with conventional transient elastography (TE), as reflected in the stronger AUROC values reported by Elkrief et al. [13] and Procopet et al. [17]. In contrast, TE-based studies such as Kumar et al. [12] and Ryu et al. [19] showed acceptable but comparatively lower performance, with broader variability in optimal cut-off thresholds. These differences may reflect both technical factors, such as depth dependency, feasibility, and measurement variability, and population-level heterogeneity, including the etiology of cirrhosis and the severity distribution of portal hypertension.

Another notable pattern is the variability in proposed stiffness thresholds for CSPH. Cut-offs ranged from approximately 15-17 kPa in some SWE cohorts to above 30 kPa in alcoholic cirrhosis populations, as observed by Ryu et al. [19]. This heterogeneity likely reflects differences in disease etiology, fibrosis architecture, inflammatory activity, and hemodynamic components of portal hypertension. Moreover, studies incorporating spleen stiffness, such as those by Jansen et al. [16] and Zykus et al. [15], suggest that splenic elastography may complement liver measurements, particularly in rule-in or sequential diagnostic algorithms. However, spleen stiffness did not consistently outperform liver stiffness, indicating that while it may add incremental value, it does not appear to replace liver-based assessment. Collectively, these patterns suggest that elastography performance is context-dependent and that standardized reliability criteria and etiology-specific thresholds may be necessary to optimize diagnostic utility.

The included studies demonstrate substantial methodological heterogeneity that likely contributes to the variability in reported diagnostic performance. Differences were observed in study design (prospective vs. retrospective), patient populations (compensated vs. advanced cirrhosis; viral, alcoholic, and NASH etiologies), elastography modality (transient elastography, point shear-wave elastography, and two-dimensional shear-wave elastography), and reliability criteria for stiffness acquisition. Furthermore, HVPG thresholds were not uniformly applied across all cohorts, with some studies distinguishing between clinically significant portal hypertension (≥10 mmHg) and severe portal hypertension using higher cut-offs. Variability in proposed stiffness thresholds (ranging from approximately 15 to >30 kPa) further limits direct comparability and suggests potential spectrum effects. Although the reference standard (HVPG) was consistently used, risk-of-bias assessment revealed moderate concerns in patient selection and index test domains in several studies, primarily due to retrospective enrollment, lack of blinding, or incomplete reporting of measurement reliability. These factors underscore the need for standardized acquisition protocols, etiology-specific cut-offs, and multicenter validation studies to improve generalizability and reduce bias in future diagnostic accuracy research.

The findings of this review support the integration of ultrasound-based elastography into non-invasive stratification pathways for portal hypertension in adults with chronic liver disease. Liver stiffness measurement demonstrates sufficient discriminatory performance to serve as an initial screening tool for clinically significant portal hypertension, particularly in compensated cirrhosis, while spleen stiffness may provide complementary hemodynamic insight when available [20,21]. Compared with invasive HVPG measurement, elastography is rapid, repeatable, widely accessible, and suitable for longitudinal monitoring, making it particularly valuable in outpatient and resource-limited settings. However, real-world implementation requires attention to operator training, adherence to reliability criteria, and recognition of etiology-specific variability in stiffness thresholds. While elastography cannot fully replace invasive hemodynamic assessment in complex or pre-transplant scenarios, current evidence supports its role as a pragmatic, non-invasive alternative for risk stratification, therapeutic decision-making, and reducing unnecessary invasive procedures within contemporary clinical practice [22].

The findings of this review are consistent with prior literature demonstrating a moderate to strong correlation between liver stiffness measurement (LSM) and hepatic venous pressure gradient (HVPG), particularly in compensated cirrhosis. Earlier single-center and multicenter studies have similarly reported AUROC values ranging from approximately 0.75 to above 0.90 for detecting clinically significant portal hypertension (CSPH), reinforcing the reliability of elastography as a non-invasive surrogate [23,24]. Our synthesis further aligns with evidence suggesting superior diagnostic discrimination with shear-wave elastography compared to conventional transient elastography, likely due to improved depth penetration and reduced technical variability. Additionally, emerging literature has highlighted the incremental value of spleen stiffness measurement (SSM), particularly when incorporated into combined diagnostic algorithms; our review supports this complementary role rather than superiority over liver stiffness alone. Overall, the present findings consolidate existing evidence while emphasizing heterogeneity in thresholds and performance across etiologies and clinical contexts [25].

This systematic review has several strengths, including the consistent use of HVPG as the reference standard, inclusion of both liver and spleen stiffness assessments, evaluation of multiple elastography modalities, and structured quality appraisal using established bias domains. The inclusion of prospective and multicenter cohorts enhances the external validity of the findings. However, important limitations must be acknowledged. Considerable heterogeneity existed in study design, patient populations, disease severity, and stiffness cut-off thresholds, limiting direct comparability and pooled interpretability. Some studies employed retrospective enrollment or lacked clear reporting of measurement reliability criteria, introducing a moderate risk of bias. Furthermore, variations in etiology distribution (viral, alcoholic, and metabolic cirrhosis) may have influenced stiffness-pressure relationships, and not all studies reported complete diagnostic metrics such as sensitivity and specificity, restricting quantitative synthesis in certain instances.

Future research should prioritize large, prospective, multicenter diagnostic accuracy studies employing standardized elastography acquisition protocols and predefined reliability criteria to minimize heterogeneity. Etiology-specific and stage-specific stiffness thresholds require further validation to optimize clinical applicability, particularly in non-alcoholic steatohepatitis and mixed-etiology populations. Comparative studies directly evaluating liver versus spleen stiffness within unified protocols would clarify their incremental diagnostic value, and combined predictive models integrating elastography with clinical and laboratory parameters may enhance risk stratification. Additionally, longitudinal studies assessing elastography’s ability to monitor dynamic changes in portal pressure and predict clinical outcomes, such as decompensation or variceal bleeding, would strengthen its role beyond diagnostic discrimination toward prognostic utility and therapeutic monitoring.

## Conclusions

Ultrasound-based elastography demonstrates consistent and clinically meaningful diagnostic accuracy for detecting HVPG-defined portal hypertension in adults with chronic liver disease, with liver stiffness measurement showing moderate to high discriminatory performance, and shear-wave techniques generally outperforming conventional transient elastography under standardized conditions. Spleen stiffness provides complementary hemodynamic information and may enhance diagnostic stratification when incorporated into combined models. Although heterogeneity in thresholds and study design limits universal cut-off application, and elastography cannot fully substitute invasive HVPG measurement, current evidence supports its role as a reliable, non-invasive tool for identifying clinically significant portal hypertension, particularly in compensated cirrhosis, thereby offering a practical strategy to reduce invasive procedures while preserving clinically relevant risk assessment.
